# Tyrosine phenol-lyase inhibitor quercetin reduces fecal phenol levels in mice

**DOI:** 10.1093/pnasnexus/pgae265

**Published:** 2024-07-04

**Authors:** Takuma Kobayashi, Shiori Oishi, Misaki Matsui, Kodai Hara, Hiroshi Hashimoto, Kenji Watanabe, Yasukiyo Yoshioka, Noriyuki Miyoshi

**Affiliations:** Graduate School of Integrated Pharmaceutical and Nutritional Sciences, University of Shizuoka, Shizuoka, 4228526, Japan; Graduate School of Integrated Pharmaceutical and Nutritional Sciences, University of Shizuoka, Shizuoka, 4228526, Japan; School of Food and Nutritional Sciences, University of Shizuoka, Shizuoka, 4228526, Japan; Department of Pharmaceutical Sciences, University of Shizuoka, Shizuoka, 4228526, Japan; Department of Pharmaceutical Sciences, University of Shizuoka, Shizuoka, 4228526, Japan; Department of Pharmaceutical Sciences, University of Shizuoka, Shizuoka, 4228526, Japan; Graduate School of Integrated Pharmaceutical and Nutritional Sciences, University of Shizuoka, Shizuoka, 4228526, Japan; School of Food and Nutritional Sciences, University of Shizuoka, Shizuoka, 4228526, Japan; Graduate School of Integrated Pharmaceutical and Nutritional Sciences, University of Shizuoka, Shizuoka, 4228526, Japan; School of Food and Nutritional Sciences, University of Shizuoka, Shizuoka, 4228526, Japan

**Keywords:** quercetin, uremic toxin, phenol, tyrosine phenol-lyase inhibitor, intestinal bacteria

## Abstract

Tyrosine phenol-lyase (TPL), which is expressed in intestinal bacteria, catalyzes the formation of phenol from the substrate L-Tyr. Bacterial metabolite phenol and the sulfate conjugate (phenyl sulfate) are known as a type of uremic toxins, some of which exert cytotoxicity. Therefore, pathologically elevated phenol and phenyl sulfate levels are strongly implicated in the etiology and outcome of uremia. In this study, we explored the inhibitory effects of dietary polyphenols on TPL-catalyzed phenol production using a TPL activity assay. Quercetin, one of the most popular polyphenols, exhibited the strongest inhibitory activity (*K_i_* = 19.9 µM). Quercetin competitively inhibited TPL, and its activity was stronger than that of a known TPL inhibitor (Tyr analog; 2-aza-Tyr, *K_i_* = 42.0 µM). Additionally, quercetin significantly inhibited phenol production in TPL-expressing bacterial cultures (*Morganella morganii* and *Citrobacter koseri*) and Tyr-rich (5%) diet-fed C57BL/6J mouse feces. Our findings suggest that quercetin is the most promising polyphenol for reducing phenol levels. Because quercetin has a low gastrointestinal absorption rate, TPL inhibition in the intestinal tract by quercetin may be an effective strategy for treating uremia.

**Significance Statement**

Many functional food ingredients that exert health-promoting effects in vivo often exhibit low bioavailability. However, when a food ingredient acts on intestinal bacteria, it does not need to be absorbed by the host; rather, it works better when not absorbed. In this study, we found that quercetin, a typical polyphenol, markedly reduced phenol (uremic toxin) production in the digestive tract by inhibiting intestinal bacterial enzymes. Direct gut bacterial enzyme and metabolism regulation may be the mechanism by which a food ingredient, such as quercetin, which has low bioavailability, shows multiple activities in host homeostasis.

## Introduction

Tyrosine phenol-lyase (TPL, also known as β-tyrosinase), belonging to the family of lyases (EC 4.1.99.2), catalyzes the production of phenol from substrate L-Tyr (1, 2). TPL employs pyridoxal 5′-phosphate (PLP, vitamin B6) as a cofactor, which forms a Schiff base with the ɛ-amino group of lysine (Lys) 257 and is further stabilized by other neighboring residues, including phenylalanine (Phe) 123 (3). PLP-dependent enzymes are involved in amino acid metabolism, including transamination and decarboxylation. Instead of the ɛ-amino group of Lys257 in TPL, the α-amino group of L-Tyr interacts with PLP and forms a Schiff base, then hydroxy benzene moiety (phenol) is released by β-elimination reaction, and pyruvate and ammonia are formed (2, 3). TPL is expressed in several intestinal bacteria, including *Citrobacter freundii*, *Klebsiella pneumoniae*, and *Morganella morganii* (4). Phenol produced by intestinal bacteria is one of the molecules responsible for the unpleasant odor of stools. Some phenols produced by intestinal bacteria are absorbed by the host and conjugated with sulfate in the liver to form phenyl sulfate (PhS), which is then excreted in the urine (5). The bacterial metabolite phenol and its conjugate PhS are known as uremic toxins (5). Recently, PhS was reported as a compound involved in diabetic kidney disease (DKD) progression (6). PhS is cytotoxic to renal podocytes, induces inflammation, and causes albuminuria (6). Our results also demonstrated that fecal phenol levels were increased in high-fat diet-fed obese and type 2 diabetic mice (7). Since phenol and PhS are toxic, pathologically increased levels of phenol and PhS are thought to be associated not only with serious diseases but also with a decline in quality of life (QOL), including nausea, dizziness, swelling, headache, appetite loss, and fatigue (8–12). Therefore, phenol production inhibition is a promising strategy to prevent several symptoms leading to severe chronic diseases. It has been reported that some L-Tyr analogs, including L-meta-Tyr and 2-aza-Tyr, competitively inhibited TPL activity *in vitro* (13, 14). Moreover, oral administration of 2-aza-Tyr significantly reduced the PhS plasma levels in DKD model *db/db* mice and improved albuminuria (6). These findings strongly suggest that TPL inhibitors may be effective in treating phenol-induced symptoms, including uremia.

Polyphenols are a large family of naturally occurring compounds found in plants, including fruits, vegetables, herbs, spices, tea, dark chocolate, and wine (15, 16). These compounds are structurally diverse and include flavonoids, phenolic acids, lignans, and stilbenes. Polyphenols are known for their antioxidant properties that help protect against environmental damage and reduce inflammation (17). They are thought to offer various health benefits, such as protection against certain cancers, cardiovascular diseases, diabetes, and neurodegenerative diseases (18). Various molecular mechanisms, including enzyme activity inhibition, have been identified in the biological activities of polyphenols. For example, quercetin inhibits human angiotensin-converting enzyme 2 (19), epigallocatechin-3-gallate inhibits matrix metalloproteases (20), and resveratrol inhibits histone deacetylases (21). However, whether polyphenols inhibit TPL activity and reduce phenol production has not yet been determined.

In this study, we developed a TPL assay system using recombinant TPL and explored the inhibitory effects of dietary polyphenols on TPL-catalyzed phenol production. Quercetin, one of the most popular polyphenols, exhibited the strongest inhibitory activity; it competitively inhibited TPL and its activity was stronger than that of 2-aza-Tyr. Additionally, the inhibitory activity of quercetin on phenol production was observed in TPL-expressing bacterial cultures and Tyr-rich diet-fed C57BL/6J mouse feces. Our findings suggest that quercetin has the highest potential to decrease phenol levels.

## Results

### TPL inhibition by polyphenols

**Fig. 1.**
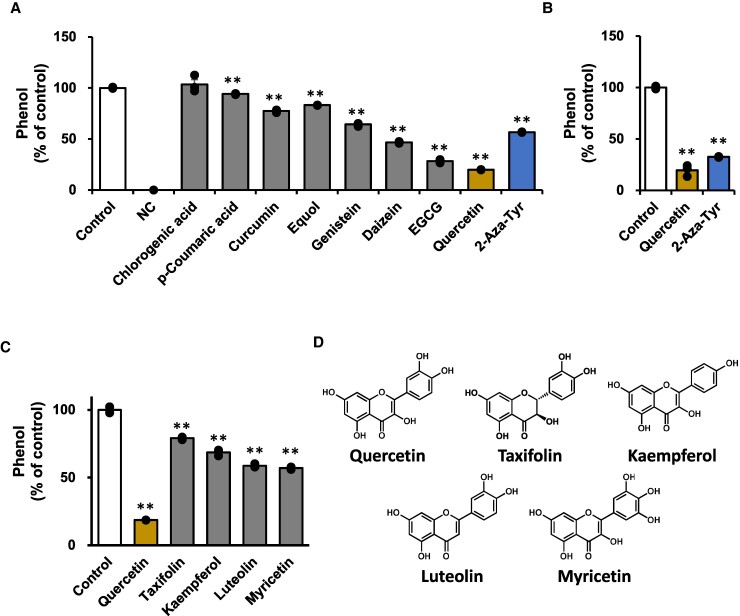
Dietary polyphenols inhibit TPL-catalyzing phenol production. A) Screening experiments of dietary polyphenols for the TPL inhibitor. A TPL assay using a recombinant TPL (JCM1672) was performed as described in the Materials and Methods section. The inhibitory activities of dietary polyphenols (500 µM) and 2-aza-Tyr (500 µM) were determined. The negative control (NC) was prepared without a substrate L-Tyr and inhibitor. B) A TPL assay using a recombinant TPL (JCM1658). The inhibitory quercetin (500 µM) and 2-aza-Tyr (500 µM) activities were determined. C) Structure–activities relationships of quercetin for TPL inhibition. A TPL assay using a recombinant TPL (JCM1672) was performed to determine the inhibitory activities of quercetin and the analogs (500 µM). D) The chemical structures of quercetin and the analogs used in this assay are shown. Data are shown by means ± SEM (*n* = 3) with individual values (black-filled circles). Statistical significance was defined as **p < 0.01.

**Fig. 2.**
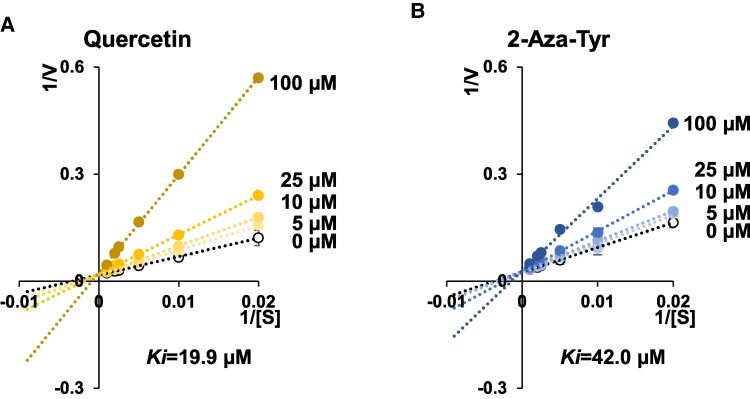
TPL enzyme kinetics. Lineweaver Burk plots of the TPL (JCM1672)-catalyzed reaction and *K_i_* value. TPL activities were determined with or without quercetin (A) or 2-aza-Tyr (B). The results are presented as the means ± SEM (*n* = 3).

To explore the inhibitor for TPL-catalyzing phenol production, we first confirmed that recombinant TPL from *M. morganii* ssp. *morganii* (JCM1672) catalyzed phenol production with *K_m_* 270 µM, *V*_max_ 52.3 µM/min, and *k*_cat_ 156.9 min^−1^, which are not very different from those reported for TPL from *C. freundii* (22). We also confirmed that 2-aza-Tyr, a known TPL inhibitor, strongly inhibited phenol production (43.4 ± 0.2%; Fig. 1A). The inhibitory activities of several dietary polyphenols were evaluated. Among eight representative dietary polyphenols, some polyphenols significantly inhibited phenol production; quercetin demonstrated the most potent activity (80.2 ± 0.1%), which was stronger than that of 2-aza-Tyr. The strongest inhibitory activity of quercetin was confirmed using another recombinant TPL isolated from *Citrobacter koseri* (JCM1658; Fig. 1B). Although structural analogs of quercetin, including taxifolin, kaempferol, luteolin, and myricetin, significantly inhibited TPL-catalyzed phenol production, the prominent activity of quercetin was observed (Fig. 1C). Additionally, quercetin inhibited TPL in a dose-dependent manner, and kinetic analysis demonstrated that its mode of inhibition was competitive, with a *K_i_* value of 19.9 µM (Fig. 2A). 2-Aza-Tyr also exhibited dose-dependent and competitive inhibition, as previously reported, with a *K_i_* value of 42.0 µM (Fig. 2B), suggesting that quercetin is a stronger inhibitor of TPL-catalyzed phenol production than 2-aza-Tyr.

### Inhibitory effect of quercetin on phenol production in bacteria

**Fig. 3.**
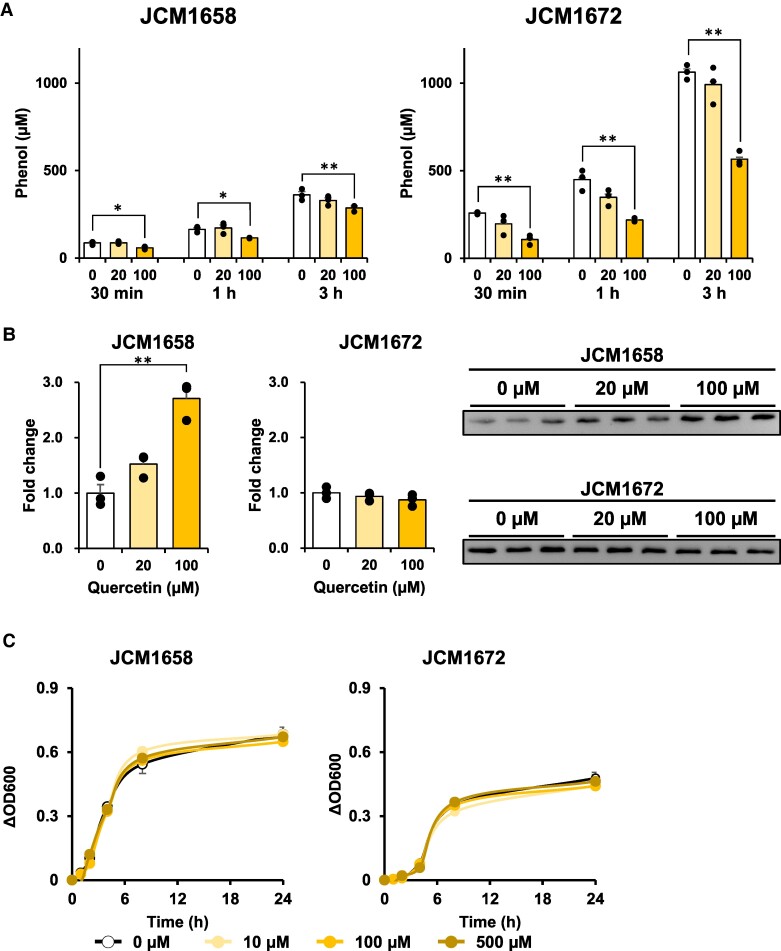
Quercetin inhibits phenol production in bacteria. A) The inhibitory effect of quercetin on phenol production in bacteria expressing TPL. Quercetin at the indicated concentration was incubated with *C. koseri* (JCM1658) and *M. morganii* ssp. *morganii* (JCM1672) suspended in an M9 salt broth supplied with 4 mM L-Tyr. Phenol levels were quantified by LC–MS. B) The effect of quercetin on TPL protein expressions in bacteria. Bacterial cell lysate after 3 h of incubation was subjected to SDS–PAGE and immunoblot analyses to determine TPL protein levels. C) The bacterial growth curves. The quercetin or DMSO (vehicle) at the indicated concentration was added to a trypticase soy broth. The bacterial growth in plastic test tubes was determined by optical density (OD_600_) using a microplate reader. Data are shown by mean ± SEM (*n* = 3) with individual values (black-filled circles). Statistical significance was defined as *p < 0.05 and **p < 0.01.

To determine the inhibitory effect of quercetin on phenol production in TPL-expressing bacteria, quercetin was exposed to *C. koseri* (JCM1658) and *M. Morganii* ssp. *morganii* (JCM1672). In these experiments, 4 mM L-Tyr was added to the bacteria to mimic concentrations in the mouse intestinal tract (23). In both bacteria, although 20 µM quercetin did not significantly affect the phenol concentration at any incubation time, 100 µM quercetin significantly reduced the phenol concentration at all incubation times, in which the maximum inhibition in JCM1658 and JCM1672 was 22.5 ± 5.4% (30 min) and 58.4 ± 6.2% (30 min), respectively (Fig. 3A). Quercetin did not decrease the TPL protein expression levels, suggesting that the reduced phenol production levels by quercetin were not due to the down-regulation of TPL (Fig. 3B). In JCM1658, although TPL protein expression was induced by quercetin, phenol levels were reduced. The weaker activity for phenol production observed in JCM1658 compared with JCM 1672 might be due to the dose-dependent elevation in TPL protein expression. Additionally, we confirmed that quercetin did not inhibit the growth of either strain (Fig. 3C), suggesting that quercetin reduces phenol production in TPL-expressing bacteria.

### Oral administration of quercetin-reduced fecal phenol levels in mice

**Fig. 4.**
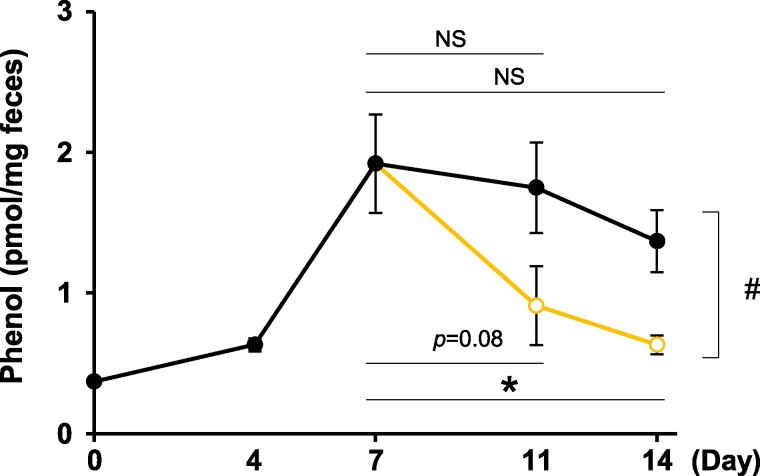
Fecal phenol levels decreased by quercetin administration. C57BL/6J mice (*n* = 12) were fed a standard diet (AIN-93G) for 1 week, followed by an L-Tyr-rich diet for 7 days to increase fecal phenol levels. The mice were then divided into two groups: a tyrosine group (*n* = 6, black circles) that continued to be fed an L-Tyr-rich diet and a quercetin group (*n* = 6, yellow open circles) that was fed an L-Tyr-rich diet containing 0.2% quercetin. Fecal samples were collected on days 0, 4, 7, 11, and 14, which were subjected to the LC–MS quantification of fecal phenol. Data are presented as mean ± SEM (*n* = 12 or 6). Statistical significance was defined as *p < 0.05 (Dunnett's test) and # (unpaired t test). NS, not significant.

The TPL inhibitory activity of quercetin was evaluated by measuring fecal phenol levels in C57BL/6J mice fed an L-Tyr-rich diet. Fecal phenol levels were significantly increased by L-Tyr-rich diet for 7 days (1.92 ± 0.35 nmol/mg feces; Fig. 4). Elevated levels of phenol induced by the L-Tyr-rich diet were significantly lowered by quercetin administration (0.63 ± 0.07 nmol/mg feces; Fig. 4). No differences in the daily food intake were observed during the experimental period (data not provided). The TPL protein expression level in mouse feces was below the detection limit.

## Discussion

In this study, we explored TPL inhibitors and found one of the dietary polyphenols, quercetin, strongly inhibited TPL activity, and reduced the phenol levels in *in vitro* and *in vivo*. Our results revealed that quercetin competitively inhibited TPL and that its inhibitory activity was stronger than that of the known TPL inhibitor, 2-aza-Tyr. These results suggested that quercetin is an effective food factor for treating several diseases and symptoms, including DKD.

Uremic toxins, represented by urea and uric acid, are a general term for waste products excreted in urine after nutrients are metabolized and filtered by the renal glomeruli (5). When urinary toxins accumulate in the blood owing to impaired renal function, they affect the heart, digestive organs, and cranial nerves, causing various uremia symptoms (nausea, dizziness, swelling, headache, loss of appetite, fatigue, etc.) (8). Over 100 metabolites have been identified and classified as uremic toxins (24–26). The toxicity of phenol, *p*-cresol, and indole has been widely studied. These amino acid–derived uremic toxins are formed via the intestinal bacteria–expressing enzymes, TPL, tyrosine lyase, and tryptophan indole-lyase (TIL), and are detected as sulfate conjugates in the host's blood and urine. Our previous study demonstrated that a GC-MS nontargeted analysis of fecal volatile compounds in lifestyle-related disease models KK-*A^y^* and C57BL/6J mice fed normal and high-fat diets identified phenol (108-fold increase) as the metabolite most significantly elevated by the interaction between “pathology (diabetes)” and “dietary content (high-fat diet)” (7). At this time, *p*-cresol (4.6-fold increase) and indole (9.4-fold increase) levels also increased, but their ratios were not as pronounced as those of phenol. Kikuchi et al. (6) identified PhS as a causative agent of diabetic nephropathy and reported that 2-aza-Tyr administration inhibited phenol production and improved nephropathy. DKD is a diabetic complication and a major cause of chronic kidney disease (CKD) (27). High-protein diets increase glomerular filtration, which, in turn, increases glomerular filtration rate (GFR) and kidney burden and decreases renal function (28). Another problem with protein intake in patients with CKD and DKD is the presence of uremic toxins. Because renal function is reduced in patients with CKD, the excretion of these compounds is difficult, leading to increased blood concentrations. This results in various metabolic disorders, such as insulin resistance, ectopic lipid dysfunction in the muscle and liver, and cytotoxicity to proximal tubular cells (29). Therefore, the latest guidelines recommend limiting protein intake for treating CKD, including DKD (30). However, patients with DKD are at high risk for sarcopenia and frailty (31) because they are elderly, and decreased muscle strength, muscle mass, and physical function are associated with increased mortality in patients with CKD (32). Therefore, there is a need to develop dietary treatments in addition to protein restriction.

Quercetin, a flavonol that is a strong TPL inhibitor, has been reported to suppress CKD progression (33). Although CKD progression suppression by quercetin has been evaluated using the urinary albumin/creatinine ratio (33), urinary protein (34), and GFR (35), no study has reported a decrease in urinary PhS and fecal phenol. In this study, we found that quercetin inhibited phenol production in *in vitro* and *in vivo*. In *in vitro* analysis, quercetin increased TPL protein expression in *C. koseri* (JCM1658; Fig. 3B). Since it has been reported that TPL expression is controlled by the level and balance of nutritional sources (2), quercetin may inhibit or promote metabolic pathways. However, TPL expression was induced only in *C. koseri* (JCM1658) but not in *M. morganii* (JCM1672), suggesting that this may be a cell-specific phenomenon. In *in vivo* experiments, we analyzed mouse feces using liquid chromatography–mass spectrometry (LC–MS) and confirmed that quercetin reaches the colon. It is well-known that quercetin absorption in the intestinal tract is very low (∼1.5–24%) (36, 37). However, for TPL inhibition, quercetin does not need to be absorbed by the host. Quercetin functions better in the intestinal tract if not absorbed. The strong activity of quercetin in *in vivo* experiment might be due to its low absorbability. Additionally, Wang et al. (38) reported that isoquercitrin (ISO), a quercetin glycoside, decreased urinary indole sulfate (IS). In contrast to the prevailing concept of TIL inhibition, ISO inhibits IS formation primarily by hindering L-Trp transport into the bacteria. Even if quercetin glycoside is taken from dietary food, it may be effective through L-Tyr transportation (38).

Generally, patients with diabetes may exhibit cracked or itchy skin. It has been pointed out that dehydration due to high blood sugar and autonomic nervous system disorders that make it difficult to sweat are some of the factors behind such skin, but Iizuka et al. reported that phenol produced by intestinal bacterial metabolism accumulates on the skin, causing skin aging such as dull and dry skin (39, 40). In contrast, the intestine (large and small intestines) is the second most neuron-rich organ after the brain, with two plexuses (Meissner' plexus, located in the submucosa and mainly involved in secretory control; Auerbach's plexus, located between the internal ring and the external longitudinal muscles of the muscularis propria and mainly controlling digestive tract movement) in the intestinal wall. Phenol is a neurotoxic chemical that is used for denervation in medical procedures. Phenols produced in the gastrointestinal tract may cause constipation and diarrhea through neurotoxicity in the gastrointestinal tract. Thus, amino acid–derived urea toxins may be deeply related not only to serious diseases such as diabetes and CKD, but also to daily QOL, including skin aging, bowel movements, and other cosmetic issues. Further analyses are necessary to clarify whether adequate amino acid–derived uremic toxin inhibition can improve uremic symptoms and contribute to QOL, whether symptoms such as skin irritation and constipation can be improved, and to what extent uremic toxin inhibition can contribute. Polyphenols are considered safe based on dietary experience and can be added to daily diets to prevent a decline in QOL.

## Materials and methods

### Reagents

Curcumin, daidzein, genistein, equol, quercetin, chlorogenic acid, *p*-coumaric acid, resveratrol, kaempferol, luteolin, taxifolin, and myricetin were purchased from the Tokyo Chemical Industry (Tokyo, Japan). (−)-Epigallocatechin gallate was obtained from Taiyo Kagaku Co. Ltd (Mie, Japan). 2-Aza-Tyr, as the TPL inhibitor, was prepared as previously described (41).

### Preparation of recombinant TPL

TPL genes from *M. morganii* ssp. *morganii* (JCM1672) and *C. koseri* (JCM1658) were inserted into the pET-15b vector. We added 0.1 mM isopropyl 1-thio-β-D-galactopyranoside to *Escherichia coli* BL21 (DE3) transformed with TPL/pET-15b cultured in an LB broth medium at 37 °C to an optical density of ∼0.8 at 660 nm, and the cells were cultured at 25 °C overnight. The cells harvested by centrifugation were resuspended in buffer I (10 mM 4-(2-hydroxyethyl)-1-piperazineethanesulfonic acid-NaOH, pH 8.0, and 50 mM NaCl). The cell lysate prepared by sonication was centrifuged for 10 min at 4 °C (20,800 × *g*). The supernatant filtered by a 0.45-µm filter was applied to a HisTrap HP column (Cytiva), and bound protein was eluted with a linear gradient of 20–500 mM imidazole. TPL fractions were applied to an anion-exchange column (HiTrap Q HP column; GE Healthcare). Bound proteins were eluted using a linear gradient of 50 mM to 1 M NaCl. The eluted protein was passed through a size-exclusion column (HiLoad 16/600 Superdex200; GE Healthcare), equilibrated with buffer II (50 mM potassium phosphate buffer [pH 8.0], 1 mM dithiothreitol, and 1 mM ethylenediaminetetraacetic acid), and concentrated to 10–15 mg/mL using a Vivaspin concentrator (Sartorius). Purified protein was frozen in liquid N_2_ and stored at −80 °C until use.

### TPL assay

The TPL assay was performed as described previously with slight modifications (22, 42). The assay mix (final volume, 40 µL) contained 50 mM potassium phosphate, pH 8.0, 50 µM PLP, 500 µM L-Tyr, 500 µM inhibitor, and 12.5 µg/mL TPL. The mixture without PLP and TPL was incubated at 37 °C for 15 min, and the reaction was initiated by adding PLP and TPL. After incubation at 37 °C for 3 min, the reaction was terminated by adding 50 µL 1 N HCl. After centrifugation at 20,800 × *g* for 10 min, the phenol content of the supernatant was determined using LC–MS. LC–MS was performed using a 1290 Infinity II LC System (Agilent Technologies, Santa Clara, CA, USA) coupled to a G6410B triple quadrupole mass spectrometer (Agilent Technologies). LC separation was conducted with an ODS-120H column (1.9 μm, 50 mm × 2.0 mm i.d., TOSOH) at 40 °C using water as solvent A and MeOH as solvent B. The gradient condition for B % was 0–4 min = 5–47.5%, 4.01–6 min = 90%, 6.01–8 min = 90–5%, and 8.01–11 min = 5%. The mobile phase flow rate was maintained at a flow phase of 0.4 mL/min. Triple quadrupole MS was performed in atmospheric-pressure chemical ionization negative mode. Compounds were detected using single ion monitoring mode. Phenol and 4-isopropyl phenol (IS for bacterial cultures and mouse fecal analysis described below) were monitored at 93.0 *m/z* and 135.0, respectively. The phenol concentration was quantified using external (recombinant TPL) and internal (bacterial cultures and mouse feces, described below) standard methods. The type of inhibition was determined using a double-reciprocal plot of the initial velocity in the presence and absence of the inhibitor. The *K_i_* values were determined using GraphPad Prism version 9.3.1 for Windows (GraphPad Software, La Jolla, CA, USA).

### Effect of quercetin on the phenol production in bacteria

*Morganella morganii* ssp. *morganii* (JCM1672) and *C. koseri* (JCM1658) were precultured in a trypticase soy broth for 24 h. Bacterial pellets were harvested by centrifugation, washed thrice with an M9 salt broth, resuspended in the same broth, and adjusted to an OD_600_ of 1.0. The suspension was then incubated with quercetin or dimethyl sulfoxide (DMSO) (vehicle) at 37 °C for 30 min, 1 h, or 3 h. Then, the suspensions were added to an equivalent volume of 50 µM 4-isopropyl phenol solution, two volumes of 2 N HCl, and six volumes of ethyl acetate and then vortexed for 10 min. The mixture was centrifuged at 5,000 × *g*, 4 °C, for 10 min, and the organic phase was collected in a new tube. The solvent was evaporated at 30 °C (EZ-2, GeneVac, UK), and the residue was resolved with 20% MeOH to be subject to LC–MS. An aliquot of the bacterial suspension was mixed with a sodium dodecyl sulfate (SDS) sample buffer and subjected to SDS–polyacrylamide gel electrophoresis (SDS–PAGE) analysis, followed by western blotting using an anti-TPL primary antibody prepared with immunized recombinant TPL (JCM1672). The density of specific bands was determined using the ImageJ image analysis software (National Institutes of Health, Bethesda, MD, USA).

### Animal experiment

All animal experimental procedures were approved by the Animal Ethics Committee of the University of Shizuoka (approval number 235413) and performed according to the National Institutes of Health Guide for the Care and Use of Laboratory Animals (NIH Publications No. 8023, revised 1978) and the University of Shizuoka guidelines for the Care and Use of Laboratory Animals. Five-week-old male C57/BL6J mice (*n* = 12) were obtained from CLEA (Tokyo, Japan). The mice were housed in plastic cages and had free access to drinking water under controlled humidity conditions (55 ± 5%), light (12-h light–dark alternating cycles), and temperature (23 ± 1 °C). After a 1-week adaptation period with a standard diet AIN-93G provided ad libitum, the mice were fed a Tyr-rich diet (5%) for 7 days to induce the phenol production in mice gut, as previously reported (39). The mice were then divided into two groups: a tyrosine group (*n* = 6) that continued to be fed an L-Tyr-rich diet and a quercetin group (*n* = 6) that was fed an L-Tyr-rich diet containing 0.2% quercetin. Food intake and body weight were monitored. Fecal samples collected on days 0, 4, 7, 11, and 14 were subjected to phenol quantification, with 100 mg feces in 10 volumes of 50 mM potassium phosphate buffer (pH 8.0) micro-destructed (5,000 rpm, 20 s × two times at 4 °C) by MicroSmash MS-100R (TOMY, Japan) with 5.0 ϕ zirconia beads (6 beads/tube). After centrifugation at 20,800 × *g* at 4 °C for 10 min, a 400-µL supernatant was mixed with 40 µL of 50 µM 4-isopropyl phenol solution, 200 µL of 6 N HCl, and 600 µL of ethyl acetate and then vortexed for 10 min. The mixture was centrifuged at 5,000 × *g*, 4 °C, for 10 min, and the organic phase was collected to a new tube. The solvent was evaporated, and the residue was resolved with 20% MeOH to be subject to LC–MS.

### Statistical analysis

Statistical analyses were performed using EZR (Saitama Medical Center, Jichi Medical University), a graphical user interface for R (The R Foundation for Statistical Computing), and data were analyzed using multiway and one-way ANOVA, followed by the Tukey honestly significant difference test or Dunnett's post hoc test and an unpaired t test. Differences were considered statistically significant at *P* < 0.05.

## Data Availability

All data are included in the manuscript.
